# Supplementary material for: Extreme environmental conditions reduce coral reef fish biodiversity and productivity
Source: Nat Commun. 2020 Jul 31;11:3832. doi: 10.1038/s41467-020-17731-2 (PMC7395083; doi:10.1038/s41467-020-17731-2)
Supplement: Supplementary file 1 — Supplementary Information [file 41467_2020_17731_MOESM1_ESM.pdf]

Extreme environmental conditions reduce coral reef fish biodiversity and productivity

Authors: Simon J. Brandl[1,2,3,4]*†, Jacob L. Johansen[5,6]†, Jordan M. Casey[3,4], Luke Tornabene[7], Renato A. Morais[8,9], John A. Burt[6]

† these authors contributed equally

***Corresponding author:**

Simon J. Brandl, simonjbrandl@gmail.com, (ORCID: 0000-0002-6649-2496)

Jacob L. Johansen, jacob.johansen@hawaii.edu, (ORCID: 0000-0002-2912-2146)

Affiliations:

1 Department of Biological Sciences, Simon Fraser University, Burnaby, BC, Canada

2 CESAB - FRB, 5 Rue de l'École de Médecine, 34000, Montpellier, France

3 PSL Université Paris: CNRS-EPHE-UPVD USR3278 CRIOBE, Université de Perpignan, Perpignan, France

4 Laboratoire d'Excellence "CORAIL," Perpignan, France

5 Hawai'i Institute of Marine Biology, University of Hawai'i at Manoa, Kane'ohe, HI, USA

6 Marine Biology Laboratory, Centre for Genomics and Systems Biology, New York University Abu Dhabi, Abu Dhabi, United Arab Emirates

7 School of Aquatic and Fishery Sciences and the Burke Museum of Natural History and Culture, University of Washington, Seattle, WA, USA

8 ARC Centre of Excellence for Coral Reef Studies, James Cook University, Townsville, QLD, Australia

9 College of Science and Engineering, James Cook University, Townsville, QLD, Australia

Supplementary Material

Location	Year	Season	Maximum	Minimum	Mean
Arabian Gulf	2012	fall	35.58	24.73	30.89
Arabian Gulf	2012	spring	32.79	27.24	30.80
Arabian Gulf	2012	summer	35.99	30.77	33.94
Arabian Gulf	2012	winter	26.52	21.10	24.11
Arabian Gulf	2013	fall	34.89	23.86	30.38
Arabian Gulf	2013	spring	31.89	22.25	26.30
Arabian Gulf	2013	summer	35.53	28.89	33.17
Arabian Gulf	2013	winter	28.89	17.32	22.36
Arabian Gulf	2014	fall	34.84	24.27	30.68
Arabian Gulf	2014	spring	33.47	21.18	27.12
Arabian Gulf	2014	summer	35.80	30.60	33.68
Arabian Gulf	2014	winter	25.40	18.65	21.25
Arabian Gulf	2015	fall	35.77	23.33	30.66
Arabian Gulf	2015	spring	32.46	20.53	26.67
Arabian Gulf	2015	summer	35.80	31.48	33.87
Arabian Gulf	2015	winter	25.99	19.34	22.33
Arabian Gulf	2016	fall	34.68	19.03	32.06
Arabian Gulf	2016	spring	32.05	22.23	25.44
Arabian Gulf	2016	summer	35.34	30.57	33.65
Arabian Gulf	2016	winter	23.28	19.20	21.25
Gulf of Oman	2012	fall	31.89	25.43	29.01
Gulf of Oman	2012	summer	34.78	23.55	29.96
Gulf of Oman	2012	winter	26.48	23.50	25.15
Gulf of Oman	2013	fall	32.64	24.27	29.31
Gulf of Oman	2013	spring	31.05	22.54	25.78
Gulf of Oman	2013	summer	33.05	24.22	29.08
Gulf of Oman	2013	winter	26.65	22.06	24.26
Gulf of Oman	2014	fall	32.90	25.02	29.83
Gulf of Oman	2014	spring	33.08	21.72	25.85
Gulf of Oman	2014	summer	33.94	24.24	30.75
Gulf of Oman	2014	winter	24.97	21.49	22.85

Supplementary Table 1: Seasonal temperature profiles derived from *in situ* data loggers deployed at various periods at the sample sites. Maximum, minimum, and mean values are daily estimates. Seasons were defined as: spring = March 1st to May 31st; summer = June 1st to August 31st; fall = September 1st to November 30th; winter = December 1st to February 28th.

Family	*Species*	*AG*	*GO*	*R*	*Reference*
Apogonidae	*Apogon coccineus*	6	10	*	present
Apogonidae	*Apogonichthyoides taeniatus*	2	0	*	present
Apogonidae	*Cheilodipterus novemstriatus*	2	9	*	present
Apogonidae	*Cheilodipterus persicus*	0	1	*	Krupp & Müller 1994
Apogonidae	*Fowleria variegata*	5	1	*	present
Apogonidae	*Ostorhinchus cyanosoma*	0	15	*	Krupp & Müller 1994
Apogonidae	*Ostorhinchus fleurieu*	0	30	*	Eagderi et al. 2019
Batrachoididae	*Colletteichthys occidentalis*	6	0	*	present
Blenniidae	*Antennablennius adenensis*	0	54	*	Bishop 2003
Blenniidae	*Ecsenius pulcher*	8	97	*	present
Blenniidae	*Laiphognathus multimaculatus*	1	0	*	present
Bythitidae	*Dinematichthys iluocoeteoides*	5	0	*	present
Gobiidae	*Asterropteryx semipunctata*	0	2	*	Krupp & Müller 1994
Gobiidae	*Callogobius bifasciatus*	2	0	*	present
Gobiidae	*Callogobius speA*	0	3	*	Eagderi et al. 2019
Gobiidae	*Coryogalops anomalus*	65	33	*	present
Gobiidae	*Eviota guttata*	0	69	*	Krupp & Müller 1994
Gobiidae	*Eviota punyit*	0	12	*	Krupp & Müller 1994[1]
Gobiidae	*Favonigobius melanobranchus*	1	0	*	present
Gobiidae	*Fusigobius inframaculatus*	0	3	*	Eagderi et al. 2019
Gobiidae	*Gnatholepis caudimaculata*	0	14	*	Eagderi et al. 2019
Gobiidae	*Gobiodon reticulatus*	0	2	*	Bishop 2003
Gobiidae	*Heteroeleotris vulgaris*	0	405	*	Eagderi et al. 2019
Gobiidae	*Istigobius decoratus*	0	15	*	Eagderi et al. 2019
Gobiidae	*Priolepis cincta*	0	4	*	Winterbottom & Burridge 1992
Gobiidae	*Priolepis randalli*	0	2	*	Winterbottom & Burridge 1993
Gobiidae	*Priolepis semidoliata*	0	10	–	NA
Gobiidae	*Trimma corallinum*	0	11	*	Eagderi et al. 2019[2]
Muraenidae	*Gymnothorax speA*	0	12	*	Eagderi et al. 2019[3]
Ostraciidae	*Ostracion cubicus*	0	3	*	Eagderi et al. 2019
Pomacanthidae	*Pomacanthus maculosus*	7	0	*	present
Pomacentridae	*Chromis flavaxilla*	0	19	*	Bishop 2003
Pomacentridae	*Chromis xanthopterygius*	0	3	*	Bishop 2003
Pomacentridae	*Neopomacentrus cyanomos*	0	38	*	Bishop 2003
Pomacentridae	*Neopomacentrus miryae*	0	38	–	NA
Pomacentridae	*Neopomacentrus sindensis*	0	6	*	Bishop 2003
Pomacentridae	*Pomacentrus aquilus*	3	0	*	present
Pomacentridae	*Pomacentrus leptus*	0	5	*	Bishop 2003
Pomacentridae	*Pomacentrus trichrourus*	5	0	*	present
Pseudochromidae	*Pseudochromis aldabraensis*	0	4	*	Bishop 2003
Pseudochromidae	*Pseudochromis linda*	1	0	*	present
Pseudochromidae	*Pseudochromis nigrovittatus*	2	1	*	present
Pseudochromidae	*Pseudochromis persicus*	1	0	*	present
Serranidae	*Cephalopholis hemistiktos*	2	2	*	present
Syngnathidae	*Corythoichthys flavofasciata*	0	5	*	Froese & Pauly 2019
Syngnathidae	*Doryrhamphus excisus*	0	3	*	Bishop 2003
Tripterygiidae	*Enneapterygius ventermaculus*	131	262	*	present
Tripterygiidae	*Helcogramma fuscopinna*	0	134	–	NA

[1]identified as *E. sebreei*
[2]synonymous with *T. winterbottomi*
[3]genus level

Supplementary Table 2: Presence, abundance, and previous records of species sampled in the present study. Each row represents a species, with columns *AG* (Arabian Gulf) and *GO* (Gulf of Oman) indicating the abundance of the species in our samples. Column *R* indicates whether the species has been previously recorded in other parts of the Arabian Gulf (* = yes, – = no). References for previous records are provided.

Population I	Population II	Estimate	LCI	UCI
C. anomolus.AG	*E. pulcher.AG*	*0.486*	*-0.079*	*1.054*
C. anomolus.AG	*E. ventermaculus.AG*	*1.360*	*0.808*	*1.949*
C. anomolus.AG	*E. pulcher.GoO*	*1.114*	*0.581*	*1.726*
C. anomolus.AG	*E. ventermaculus.GoO*	*1.633*	*0.939*	*2.342*
C. anomolus.AG	*E. guttata.GoO*	*1.143*	*0.534*	*1.759*
C. anomolus.AG	*H. fuscopinna.GoO*	*2.392*	*1.758*	*2.992*
C. anomolus.AG	*H. vulgaris.GoO*	*0.492*	*-0.061*	*1.078*
E. pulcher.AG	*E. ventermaculus.AG*	*0.879*	*0.509*	*1.252*
E. pulcher.AG	*E. pulcher.GoO*	*0.636*	*0.244*	*1.016*
E. pulcher.AG	*E. ventermaculus.GoO*	*1.159*	*0.624*	*1.737*
E. pulcher.AG	*E. guttata.GoO*	*0.656*	*0.227*	*1.134*
E. pulcher.AG	*H. fuscoguttata.GoO*	*1.905*	*1.463*	*2.341*
E. pulcher.AG	*H. vulgaris.GoO*	*0.011*	*-0.368*	*0.417*
E. ventermaculus.AG	*E. pulcher.GoO*	*-0.245*	*-0.640*	*0.118*
E. ventermaculus.AG	*E. ventermaculus.GoO*	*0.277*	*-0.260*	*0.815*
E. ventermaculus.AG	*E. guttata.GoO*	*-0.225*	*-0.680*	*0.212*
E. ventermaculus.AG	*H. fuscopinna.GoO*	*1.024*	*0.578*	*1.449*
E. ventermaculus.AG	*H. vulgaris.GoO*	*-0.878*	*-1.265*	*-0.508*
E. pulcher.GoO	*E. ventermaculus.GoO*	*0.519*	*-0.0290*	*1.073*
E. pulcher.GoO	*E. guttata.GoO*	*0.020*	*-0.426*	*0.494*
E. pulcher.GoO	*H. fuscopinna.GoO*	*1.274*	*0.839*	*1.726*
E. pulcher.GoO	*H. vulgaris.GoO*	*-0.628*	*-1.037*	*-0.253*
E. ventermaculus.GoO	*E. guttata.GoO*	*-0.502*	*-1.125*	*0.106*
E. ventermaculus.GoO	*H. fuscopinna.GoO*	*0.750*	*0.130*	*1.344*
E. ventermaculus.GoO	*H. vulgaris.GoO*	*-1.148*	*-1.710*	*-0.584*
E. guttata.GoO	*H. fuscopinna.GoO*	*1.252*	*0.735*	*1.778*
E. guttata.GoO	*H. vulgaris.GoO*	*-0.647*	*-1.094*	*-0.148*
H. fuscopinna.GoO	*H. vulgaris.GoO*	*-1.906*	*-2.363*	*-1.449*

Supplementary Table 3: Contrasts between levels of the explanatory variable for the model testing CT_{max} differences in cryptobenthic reef fishes. Population columns highlight the contrast estimated in the model, whereas the estimate and its confidence intervals indicate estimated differences.

**Population I**	**Population II**	**Estimate**	**LCI**	**UCI**
C. anomolus.AG	*E. pulcher.AG*	*0.613*	*0.173*	*1.069*
C. anomolus.AG	*E. ventermaculus.AG*	*-0.400*	*-0.851*	*0.054*
C. anomolus.AG	*E. pulcher.GoO*	*0.747*	*0.316*	*1.211*
C. anomolus.AG	*E. ventermaculus.GoO*	*-1.391*	*-1.887*	*-0.888*
C. anomolus.AG	*E. guttata.GoO*	*-0.784*	*-1.241*	*-0.317*
C. anomolus.AG	*H. fuscopinna.GoO*	*-1.235*	*-1.736*	*-0.754*
C. anomolus.AG	*H. vulgaris.GoO*	*-0.080*	*-0.549*	*0.384*
E. pulcher.AG	*E. ventermaculus.AG*	*-1.011*	*-1.313*	*-0.709*
E. pulcher.AG	*E. pulcher.GoO*	*0.137*	*-0.165*	*0.446*
E. pulcher.AG	*E. ventermaculus.GoO*	*-2.003*	*-2.402*	*-1.641*
E. pulcher.AG	*E. guttata.GoO*	*-1.394*	*-1.704*	*-1.076*
E. pulcher.AG	*H. fuscopinna.GoO*	*-1.847*	*-2.206*	*-1.489*
E. pulcher.AG	*H. vulgaris.GoO*	*-0.694*	*-1.010*	*-0.358*
E. ventermaculus.AG	*E. pulcher.GoO*	*1.149*	*0.847*	*1.459*
E. ventermaculus.AG	*E. ventermaculus.GoO*	*-0.990*	*-1.382*	*-0.610*
E. ventermaculus.AG	*E. guttata.GoO*	*-0.381*	*-0.706*	*-0.065*
E. ventermaculus.AG	*H. fuscopinna.GoO*	*-0.836*	*-1.201*	*-0.475*
E. ventermaculus.AG	*H. vulgaris.GoO*	*0.318*	*-0.016*	*0.648*
E. pulcher.GoO	*E. ventermaculus.GoO*	*-2.138*	*-2.526*	*-1.766*
E. pulcher.GoO	*E. guttata.GoO*	*-1.530*	*-1.843*	*-1.213*
E. pulcher.GoO	*H. fuscopinna.GoO*	*-1.985*	*-2.341*	*-1.615*
E. pulcher.GoO	*H. vulgaris.GoO*	*-0.832*	*-1.174*	*-0.519*
E. ventermaculus.GoO	*E. guttata.GoO*	*0.607*	*0.231*	*1.018*
E. ventermaculus.GoO	*H. fuscopinna.GoO*	*0.152*	*-0.260*	*0.582*
E. ventermaculus.GoO	*H. vulgaris.GoO*	*1.307*	*0.895*	*1.691*
E. guttata.GoO	*H. fuscopinna.GoO*	*-0.453*	*-0.822*	*-0.088*
E. guttata.GoO	*H. vulgaris.GoO*	*0.700*	*0.360*	*1.041*
H. fuscopinna.GoO	*H. vulgaris.GoO*	*1.153*	*0.799*	*1.543*

Supplementary Table 4: Contrasts between levels of the explanatory variable for the model testing CT_{min} differences in cryptobenthic reef fishes. Population columns highlight the contrast estimated in the model, whereas the estimate and its confidence intervals indicate estimated differences.

**Trials**	**Location**	**Species**	**N**
CT_{max}			
	AG	*Coryogalops anomolus*	3
	AG	*Ecsenius pulcher*	10
	AG	*Enneapterygius ventermaculus*	12
	GoO	*Ecsenius pulcher*	10
	GoO	*Enneapterygius ventermaculus*	3
	GoO	*Eviota guttata*	6
	GoO	*Helcogramma fuscopinna*	6
	GoO	*Heterelelotris vulgaris*	10
CT_{min}			
	AG	*Coryogalops anomolus*	3
	AG	*Ecsenius pulcher*	11
	AG	*Enneapterygius ventermaculus*	10
	GoO	*Ecsenius pulcher*	10
	GoO	*Enneapterygius ventermaculus*	5
	GoO	*Eviota guttata*	9
	GoO	*Helcogramma fuscopinna*	6
	GoO	*Heterelelotris vulgaris*	8

Supplementary Table 5: Sample sizes for CT-trials across species and locations. While sample sizes are low for some species, CT-trials have been shown to yield highly reproducible and taxonomically preserved results.

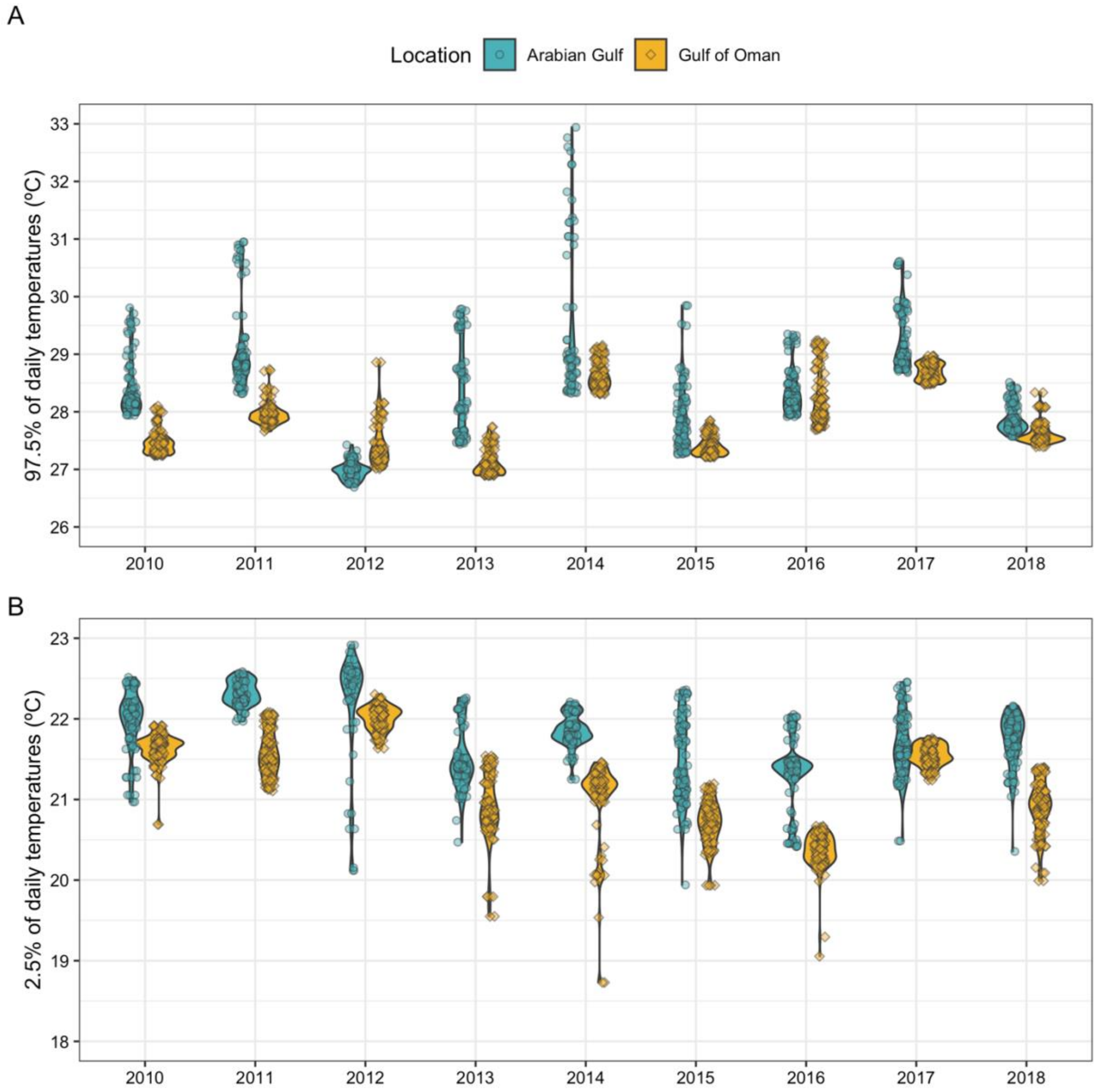

Supplementary Figure 1: 97.5% and 2.5% quantiles of remotely sensed temperatures from each year between 2010 and 2018 across our sites in the Arabian Gulf (blue) and Gulf of Oman (gold). Data were sourced from the MODIS-Aqua database.

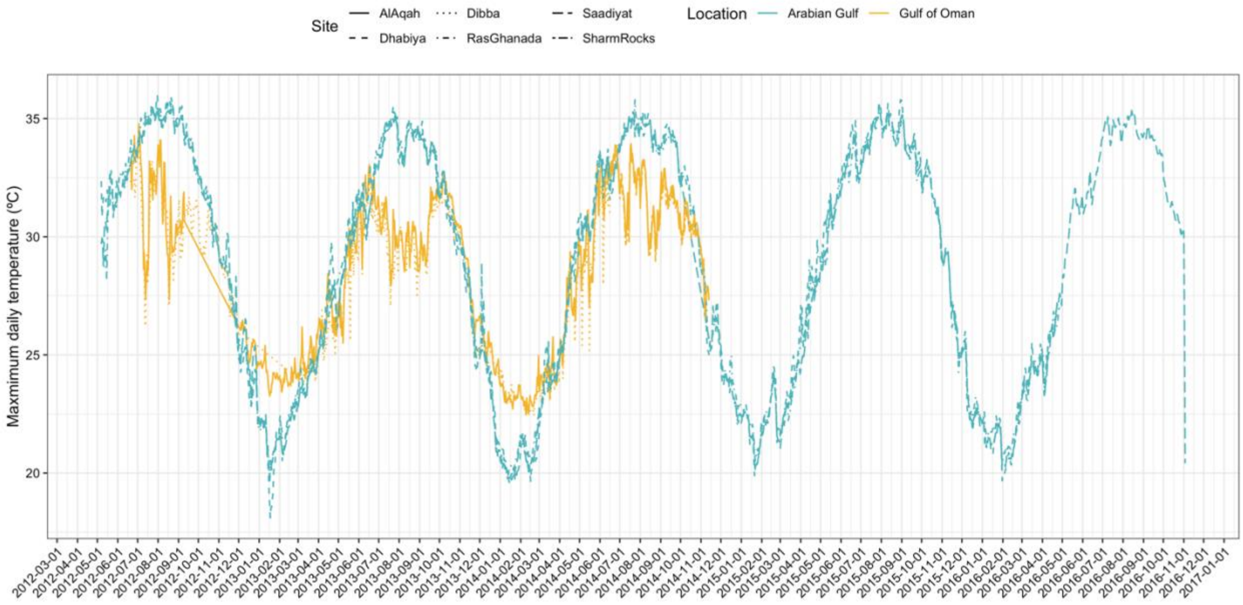

Supplementary Figure 2: Temperature records from *in situ* data loggers deployed during various time periods at the six sites across the two locations. Al Aqah = Snoopy Rock.

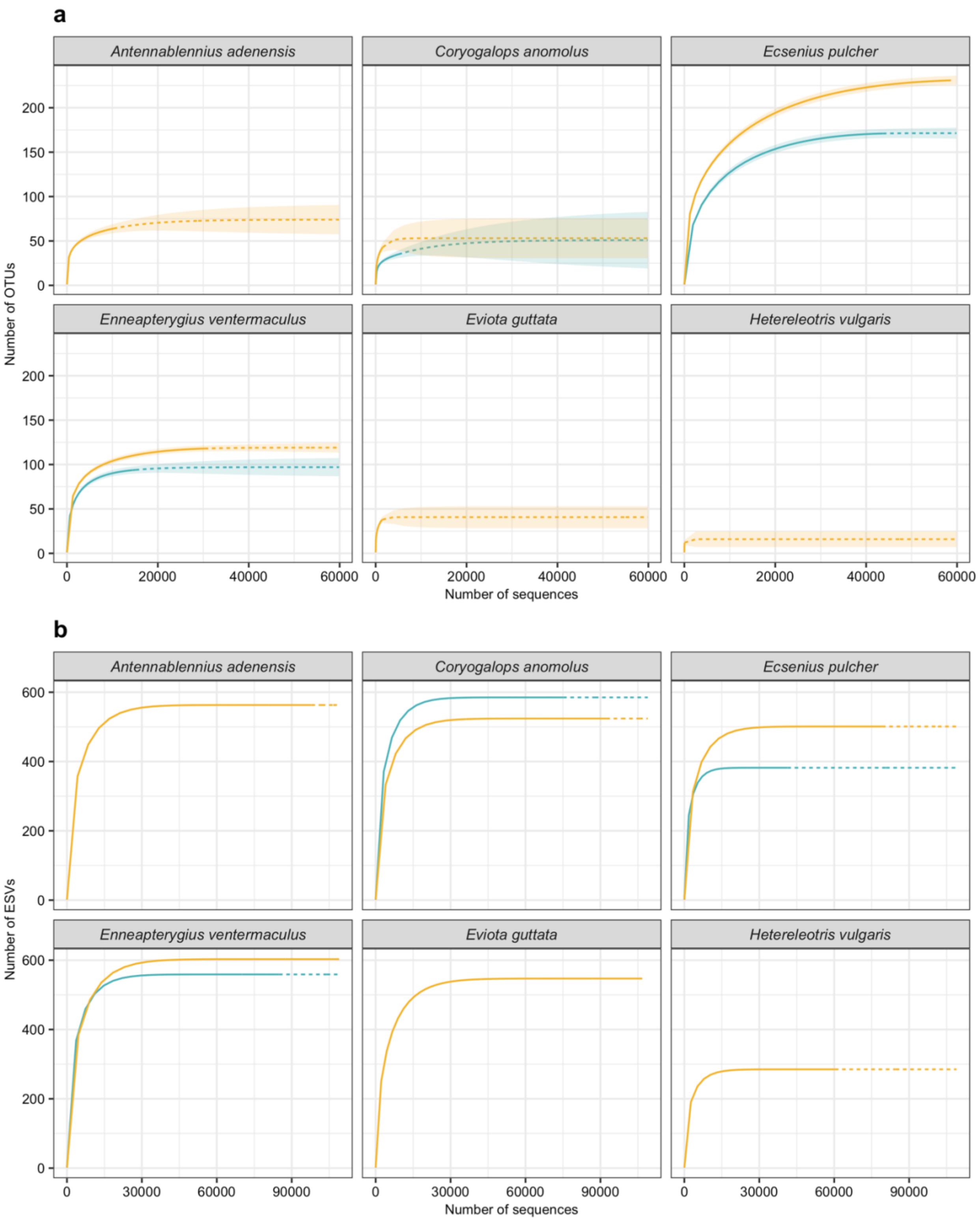

Supplementary Figure 3: Rarefaction curves of OTU and ESV richness across total sequences for six species in the Arabian Gulf (blue) and Gulf of Oman (gold). (a) OTU rarefaction curves indicate the diversity of prey items for each species and population as obtained

from gut content DNA metabarcoding with the COI marker, while (b) ESV curves show the diversity of prey items obtained with the 23S marker. Solid lines indicate interpolated richness, while dashed lines indicate extrapolated richness (to the maximum number of sequences across species). Shaded ribbons indicate 95% confidence intervals of extrapolations.
