# Supplementary material for: Extreme environmental conditions reduce coral reef fish biodiversity and productivity
Source: Nat Commun. 2020 Jul 31;11:3832. doi: 10.1038/s41467-020-17731-2 (PMC7395083; doi:10.1038/s41467-020-17731-2)
Supplement: Supplementary file 3 — Reporting Summary [file 41467_2020_17731_MOESM3_ESM.pdf]

nature research

Corresponding author(s): Simon J. Brandl, Jacob L. Johansen

Last updated by author(s): Jul 3, 2020

Reporting Summary

Nature Research wishes to improve the reproducibility of the work that we publish. This form provides structure for consistency and transparency in reporting. For further information on Nature Research policies, see our Editorial Policies and the Editorial Policy Checklist.

Statistics

For all statistical analyses, confirm that the following items are present in the figure legend, table legend, main text, or Methods section.

n/a	Confirmed	
☐	☒	The exact sample size (*n*) for each experimental group/condition, given as a discrete number and unit of measurement
☐	☒	A statement on whether measurements were taken from distinct samples or whether the same sample was measured repeatedly
☐	☒	The statistical test(s) used AND whether they are one- or two-sided<br>*Only common tests should be described solely by name; describe more complex techniques in the Methods section.*
☐	☒	A description of all covariates tested
☐	☒	A description of any assumptions or corrections, such as tests of normality and adjustment for multiple comparisons
☐	☒	A full description of the statistical parameters including central tendency (e.g. means) or other basic estimates (e.g. regression coefficient) AND variation (e.g. standard deviation) or associated estimates of uncertainty (e.g. confidence intervals)
☒	☐	For null hypothesis testing, the test statistic (e.g. *F*, *t*, *r*) with confidence intervals, effect sizes, degrees of freedom and *P* value noted<br>*Give P values as exact values whenever suitable.*
☐	☒	For Bayesian analysis, information on the choice of priors and Markov chain Monte Carlo settings
☐	☒	For hierarchical and complex designs, identification of the appropriate level for tests and full reporting of outcomes
☒	☐	Estimates of effect sizes (e.g. Cohen's *d*, Pearson's *r*), indicating how they were calculated

Our web collection on statistics for biologists contains articles on many of the points above.

Software and code

Policy information about availability of computer code

Data collection	We used a grid of points created in Adobe Illustrator to perform the analysis of benthic photographs.
Data analysis	R: code written by corresponding author (SJB), available on figshare (https://figshare.com/projects/Cryptobenthic_fish_assemblages_in_the_United_Arab_Emirates/81644) and GitHub (https://github.com/simonjbrandl/UAE18-crypto-communities).<br>Additional software was used by Jonah Ventures (Boulder, Colorado, USA) in the bioinformatic processing of gut content DNA metabarcoding samples (QIIME, UPARSE)

For manuscripts utilizing custom algorithms or software that are central to the research but not yet described in published literature, software must be made available to editors and reviewers. We strongly encourage code deposition in a community repository (e.g. GitHub). See the Nature Research guidelines for submitting code & software for further information.

Data

Policy information about availability of data

All manuscripts must include a data availability statement. This statement should provide the following information, where applicable:

- Accession codes, unique identifiers, or web links for publicly available datasets
- A list of figures that have associated raw data
- A description of any restrictions on data availability

All raw data necessary to reproduce the results are available along with raw photographs and temperature data on figshare (https://figshare.com/projects/Cryptobenthic_fish_assemblages_in_the_United_Arab_Emirates/81644). Raw data is found in Figs. 1–6 as well as Supplementary Figs 1–3. Temperature data was sourced from MODIS-Aqua (https://oceandata.sci.gsfc.nasa.gov/MODIS-Aqua/Mapped/Daily/4km/sst). GenBank was accessed as a reference database for taxonomic assignments (https://www.ncbi.nlm.nih.gov/genbank/).

Field-specific reporting

Please select the one below that is the best fit for your research. If you are not sure, read the appropriate sections before making your selection.

- [ ] Life sciences
- [ ] Behavioural & social sciences
- [x] Ecological, evolutionary & environmental sciences

For a reference copy of the document with all sections, see nature.com/documents/nr-reporting-summary-flat.pdf

Ecological, evolutionary & environmental sciences study design

All studies must disclose on these points even when the disclosure is negative.

Study description

• We tested differences in small, bottom-dwelling ('cryptobenthic') fish assemblages across two locations, the extremely hot and variable southeastern Arabian Gulf and the thermally-moderate northwester Gulf of Oman. Initial comparisons were based on in situ collections of entire communities at three distinct reefs in both locations, with three samples taken per reef (total N = 18).
• We then tested temperature tolerances of species occurring in both locations and the environmentally-benign Gulf of Oman only using critical thermal tolerance trials. For cold tolerance trials, we tested 62 individuals across six species and for heat tolerance trials, we tested 60 individuals across the same six species.
• We also examined the prey ingestion of a subset of individuals obtained from the community-samples in both locations using a DNA metabarcoding approach of tissues retained in the gastro-intestinal tract. Specifically, we tested 88 individuals across six species using both cytochrome oxidase I (COI) and 23S primers.
• We also tested for differences in body condition (indicated by length-weight relationships) in populations of three species across the two locations. For this analysis, we included all individuals obtained from the community samples and individual collections, resulting in 394 (Enneapterygius ventermaculus), 148 (Coryogalops anomolus) and 245 (Ecsenius pulcher) samples, respectively.

Research sample

A list of all species present in the sampled communities is provided as a Supplementary Table 1. Specific test (physiological and DNA metabarcoding) and analyses were performed on samples of:
• Ecsenius pulcher (Blenniidae – Arabian Gulf population)
• Ecsenius pulcher (Blenniidae – Gulf of Oman population)
• Coryogalops anomolus (Gobiidae – Arabian Gulf population)
• Coryogalops anomolus (Gobiidae – Gulf of Oman population)
• Enneapterygius ventermaculus (Tripterygiidae – Arabian Gulf population)
• Enneapterygius ventermaculus (Tripterygiidae – Gulf of Oman population)
• Antennablennius adenensis (Blenniidae – Gulf of Oman population)
• Eviota guttata (Gobiidae – Gulf of Oman population)
• Heterеleotris vulgaris (Gobiidae – Gulf of Oman population)
• Helcogramma fuscopinna (Tripterygiidae – Gulf of Oman population)

These sample species were chosen due to their abundance and availability from the studied locations as well as their ecological comparability. Samples represent populations of a given species in a given location.

Sampling strategy

Sampling procedure: First, we performed community based samples as described below. After that, we used roving diver collections to collect live individuals for physiological trials. Samples for diet metabarcoding were obtained from community samples. For community samples, we determined sufficient coverage of samples by assessing rarefaction curves, arriving at the employed sample size. For critical temperature
trials, we obtained the greatest numbers possible. Low sample sizes are taken into account in the statistical approach implemented, preventing false conclusions due to limited sample coverage. For DNA gut content metabarcoding, sample size was determined by costs of the molecular analyses; however, we again assessed adequacy of the number of samples by examining rarefaction curves of prey sequences found across the sampled population (Supplementary Figure 1).

Data collection

Community samples were obtained in the field using 3-4 SCUBA divers (SJB, JMC, JLJ, and LT) and a technique described in detail by Brandl et al. 2019 (Science). All samples obtained were processed in the evening of collection in the laboratory. All temperature trials were performed by JLJ in. the facilities of NYUAD. All laboratory work for sample preparation for DNA gut content metabarcoding (dissection and DNA extraction) was performed by SJB and LT, while post-extraction laboratory processing and bioinformatics were performed by Jonah Ventures (Boulder, CO; https://jonahventures.com/about/).

Timing and spatial scale

All field samples were collected between April 24th and May 8th 2018. Physiological trials took place betweenMay8th and May 12th. Molecular analyses were initiated in February 2019, using the preserved specimens. The spatial extent of the collections includes the following sites:
Arabian Gulf:
Dhabiya: 476 24.36383º, 54.10121º
Ras Ghanada: 24.84743º, 54.69235º
Saadiyat: 24.65771º, 477 54.48691º
Gulf of Oman:
Dibba Rock: 25.55378º, 56.35694º
Sharm Rock: 25.48229º, 480 56.36695º
Snoopy Rock: 25.49210º, 56.36401º

The timing and extent of sampling was dictated by access to field sites, funding, and time.

Data exclusions	One data point was excluded from the length-weight relationship analysis of Coryogalops anomolus (n = 149) due to an unrealistic weight estimate that must have been falsely recorded. The exclusion is clearly highlighted in the accompanying script.
Reproducibility	We undertook no attempts to reproduce the results by repeating data collection. We have ensured full reproducibility of the data analysis and provide fully accessible code and data to allow reproduction of our findings.
Randomization	Individual fishes were chosen randomly from holding tanks for the physiological trials. For the molecular analyses, we chose individuals to maximize spatial spread across the samples and with preference for the largest animals to ensure sampling of adult specimens.
Blinding	No blinding was performed for field data as the field collections are not subject to observer bias. For respirometry, no blinding was possible since the work was performed by a single individual (JLJ) and fishes were separated in their holding tanks based on therir source location.

Did the study involve field work? ☒ Yes ☐ No

Field work, collection and transport

Field conditions	Conditions were benign and constant throughout the sampled period. No specific assessments of environmental factors during sampling were performed, but water temperatures (the most relevant environmental factor for this study) varied between 27ºC and 29ºC throughout the performance of the study.
Location	Arabian Gulf:<br>Dhabiya: 476 24.36383º, 54.10121º<br>Ras Ghanada: 24.84743º, 54.69235º<br>Saadiyat: 24.65771º, 477 54.48691º<br>Gulf of Oman:<br>Dibba Rock: 25.55378º, 56.35694º<br>Sharm Rock: 25.48229º, 480 56.36695º<br>Snoopy Rock: 25.49210º, 56.36401º<br>All samples were taken between 1 and 10 meters depth.
Access & import/export	All collections were performed with approval from the Environment Agency Abu Dhabi (TMBS/18/L/179) and Dibba Municipality 1084 (unnumbered). Sample export to the University of Washington was performed under the UAE Ministry of Environment and Climate Change tissue export permit (AUD-Q-22-1110520).
Disturbance	No detectable disturbance was caused by the study.

Reporting for specific materials, systems and methods

We require information from authors about some types of materials, experimental systems and methods used in many studies. Here, indicate whether each material, system or method listed is relevant to your study. If you are not sure if a list item applies to your research, read the appropriate section before selecting a response.

Materials & experimental systems

n/a	Involved in the study	
☒	☐	Antibodies
☒	☐	Eukaryotic cell lines
☒	☐	Palaeontology and archaeology
☐	☒	Animals and other organisms
☒	☐	Human research participants
☒	☐	Clinical data
☒	☐	Dual use research of concern

Methods

n/a	Involved in the study	
☒	☐	ChIP-seq
☒	☐	Flow cytometry
☒	☐	MRI-based neuroimaging

Animals and other organisms

Policy information about studies involving animals; ARRIVE guidelines recommended for reporting animal research

Laboratory animals	The study did not use laboratory animals.
Wild animals	A full list of species sampled in the course of the study is provided in Supplementary Table 2. All individuals were either euthanized immediately after collection or transported in enclosed containers with oxygen supply to the animal husbandry facilities at NYUAD, where they were kept until trials were run. Individuals were euthanized using a clove-oil overdose immediately after conclusion of the trials to permit measurement and weighing.

Field-collected samples	A full list of species sampled in the course of the study is provided in Supplementary Table 2. All individuals were either euthanized immediately after collection or transported in enclosed containers with oxygen supply to the animal husbandry facilities at NYUAD, where they were kept until trials were run. Individuals were euthanized using a clove-oil overdose immediately after conclusion of the trials to permit measurement and weighing. Fishes were housed in appropriately sized aquaria during their time in the NYUAD aquarium facilities, using water with ambient conditions regarding temperature, salinity, etc.
Ethics oversight	All work involving live fishes was performed under NYUAD IACUC approval 18-0003.

Note that full information on the approval of the study protocol must also be provided in the manuscript.
